# Versorgungssituation des Systemischen Lupus Erythematodes in Rheinland-Pfalz und dem Saarland

**DOI:** 10.1007/s00393-024-01491-1

**Published:** 2024-03-20

**Authors:** Ciaran Alberti, Matthias Dreher, Konstantinos Triantafyllias, Andreas Schwarting

**Affiliations:** 1 Schwerpunkt Rheumatologie und klinische Immunologie, Universitätsmedizin, Johannes Gutenberg-Universität Mainz, Langenbeckstraße 1, 55131 Mainz, Deutschland; 2 RZ Rheumakliniken Rheinland-Pfalz GmbH, Bad Kreuznach, Deutschland; 3 Universitäres Centrum für Autoimmunität, Universitätsmedizin, Johannes Gutenberg-Universität Mainz, Mainz, Deutschland

**Keywords:** Systemischer Lupus erythematodes (SLE), Epidemiologie, Therapie, Patientenversorgung, Symptomatik, Systemic Lupus Erythematosus (SLE), Epidemiology, Therapy, Patient care, Symptoms

## Abstract

**Hintergrund:**

Der systemische Lupus erythematodes (SLE) ist eine klinisch heterogen verlaufende Autoimmunerkrankung, die mit hohem Leid für die Betroffenen sowie hohen sozioökonomischen Kosten verbunden ist. Eine frühe Diagnosestellung und eine adäquate medizinische Versorgung sind essenziell für einen milden Krankheitsverlauf. Es fehlen jedoch aktuelle Zahlen und Daten über die Versorgungssituation der Erkrankten in der Fläche.

**Methodik:**

Es wurden insgesamt 1546 Hausärzte, Rheumatologen, Neurologen, Nephrologen und Dermatologen in Rheinland-Pfalz und dem Saarland per Fax oder Mail mithilfe eines Fragebogens bezüglich Epidemiologie, Symptomatik, Therapie und Therapieerfolg befragt. Zusätzlich gab es die Möglichkeit, Verbesserungsvorschläge zu unterbreiten.

**Ergebnisse:**

Fünf von sechs der rückgemeldeten 635 SLE-Patienten sind weiblich. Die häufigsten Hauptsymptome waren Arthralgien, Fatigue, Myalgien und Hautveränderungen. Von den Patienten erhielten 68 % Antimalariamittel (AMM), während 46 % mit Glukokortikoiden (GC) und 50 % mit einem Immunsuppressivum (IS), v. a. Methotrexat (MTX), behandelt wurden. An Komorbiditäten litten die Patienten vor allem unter kardiovaskulären Erkrankungen, dem Fibromyalgiesyndrom und Depressionen. Rheumatologen beschrieben zudem häufig Anämien, Diabetes mellitus und Osteoporose.

**Diskussion:**

Verglichen mit den Empfehlungen der Leitlinien fiel insbesondere bei nicht rheumatologisch betreuten Patienten die geringe Quote an AMM in der Therapie auf (35 % im Mittel im Vergleich zu 81 % bei Rheumatologen). Auch (dauerhaft) hohe GC-Dosen entsprechen nicht den Empfehlungen der Literatur. Im Freitextfeld wurden vor allem mehr niedergelassene Rheumatologen und eine schnellere Terminvergabe sowie eine bessere Kommunikation und Vernetzung gewünscht. Zudem wurde häufig der Wunsch nach mehr Fortbildung und Aufklärung geäußert.

## Abstract (en)

**Background:**

Systemic lupus erythematosus (SLE) is a clinically heterogeneous autoimmune disease that is associated with great suffering for those affected, as well as high socioeconomic costs. Early diagnosis and adequate medical care are essential for a mild course of the disease. However, there is a lack of current figures and data on the care situation of patients in the area.

**Methodology:**

A total of 1546 general practitioners, rheumatologists, neurologists, nephrologists and dermatologists in Rhineland-Palatinate and Saarland were interviewed by fax or mail using a questionnaire regarding epidemiology, symptoms, therapy and therapy success. In addition, there was the possibility of making suggestions for improvement.

**Results:**

Five out of six of the 635 reported SLE patients were female. The most common main symptoms were arthralgia, fatigue, myalgia, and skin changes. Of the patients, 68% received antimalarials (AMs), whereas 46% were treated with glucocorticoids (GCs) and 50% with an immunosuppressant (IS), mainly methotrexate. In terms of comorbidities, patients suffered mainly from cardiovascular disease, fibromyalgia syndrome and depression. Rheumatologists also frequently described anaemia, diabetes mellitus and osteoporosis.

**Discussion:**

Compared with guideline recommendations, the low rate of AMs in therapy was particularly striking in patients not treated by rheumatologists (35% on average compared with 81% for rheumatologists). Additionally, (sustained) high doses of GCs are not in line with literature recommendations. In the free text field, the main requests were for more rheumatologists in private practice and faster appointment scheduling, as well as better communication and networking. In addition, the desire for more training and education was frequently expressed..

## Kurze Hinführung zum Thema

Der systemische Lupus erythematodes (SLE) ist eine klinisch heterogen verlaufende Autoimmunerkrankung, die mit hohem Leid für die Betroffenen sowie hohen sozioökonomischen Kosten verbunden ist. Obwohl hinreichend belegt ist, dass eine frühe Diagnosestellung und eine adäquate medizinische Versorgung essenziell für einen milden Krankheitsverlauf sind, gibt es weder eine ausreichende Zahl an Rheumatologen in der Fläche noch aktuelle Zahlen und Daten über die Versorgungssituation der Erkrankten in Deutschland. In der vorliegenden Arbeit wurden Daten über die Versorgungssituation des SLE in Rheinland-Pfalz und dem Saarland erfasst.

## Hintergrund und Fragestellung

Theoretisch kann der SLE jedes beliebige Organ befallen. Er gilt als die „wahrscheinlich klinisch und serologisch vielfältigste aller autoimmunen rheumatischen Erkrankungen“ [14]. Patienten^1^ beklagen häufig Arthritiden (etwa 90 %), Hauterscheinungen (80 %) oder Fieber (78 %), wobei die in etwa 50 % der Fälle auftretende sogenannte Lupusnephritis mit sekundärem chronischem Nierenversagen oft verlaufs- und prognosebestimmend ist [17, 19, 27]. Die Fatigue, eine Form der übermäßigen chronischen Müdigkeit, stellt mit einer Prävalenz von bis zu 92 % der Lupuspatienten eines der häufigsten und belastendsten Symptome dar [6, 20]. Die Variabilität des SLE erschwert oft die Diagnose und verzögert die Therapie, was wiederum zu einem längeren und häufig schwereren Leidensweg der Betroffenen führt [9, 24].

In einer Analyse von Krankenkassendaten konnten Schwarting et al. unter 3 Mio. Versicherten eine zunehmende Prävalenz von 0,056 % (zuletzt im Jahr 2014) detektieren. Gemäß einer Übersichtsstudie von Albrecht et al. schwankten die Prävalenzen zwischen 0,037–0,14 % [1, 26]. Frauen sind bis zu neunmal so häufig betroffen wie Männer [2, 8]. Die Erkrankung geht fast immer mit einer stark eingeschränkten Lebensqualität sowie in den meisten Fällen mit einer eingeschränkten Lebenserwartung einher [5, 16]. Obwohl in den letzten Jahrzehnten große Fortschritte im Verständnis und in der Therapie des SLE erzielt wurden, ist die gesundheitsbezogene Lebensqualität von SLE-Patienten heute so gering wie die von Patienten mit koronarer Herzkrankheit oder endgradiger COPD [16].

Die Ursache des SLE ist jedoch nicht vollständig verstanden, seine Ätiologie ist multifaktoriell [28].

Als Basistherapie hat sich das Antimalariamittel (AMM) Hydroxychloroquin (HCQ) etabliert, das bis auf wenige Ausnahmen jedem SLE-Patienten empfohlen wird [13]. In der Akutbehandlung spielen Glukokortikoide (GC) weiterhin eine zentrale Rolle [22]. Um langfristig GC einzusparen, können Immunsuppressiva (IS) wie Azathioprin (AZA), Mycophenolat-Mofetil (MMF) oder Methotrexat (MTX) verwendet werden [25]. Zwei Biologika sind als Zusatztherapie des SLE zugelassen (Belimumab seit 2011 und Anifrolumab seit 2022).

Die 10-Jahres-Überlebensrate von Lupuspatienten beträgt heute über 90 %, wobei in den ersten Erkrankungsjahren vor allem Infektionen als Folge der Immunsuppression durch Erkrankung und Therapie eine Rolle spielen, bis sie nach etwa fünf Jahren von den kardiovaskulären Komplikationen abgelöst werden – der akkumulierte chronische Schaden übersteigt die Krankheitsaktivität [5].

Was die Versorgung von SLE-Patienten angeht, herrscht in Deutschland eine massive Unterversorgung mit Rheumatologen. Laut Berechnungen fehlen 45 % der benötigten niedergelassenen Rheumatologen sowie 17,5 % der benötigten rheumatologischen Betten [29]. Trotz hoher individueller und gesellschaftlicher Bürden des Krankheitsbildes SLE herrscht ein Mangel an belastbaren Daten bezüglich der Versorgungsforschung im Allgemeinen sowie speziell in Deutschland. Da internationale Forschungsergebnisse oft nicht ohne Weiteres von einem Land auf das andere übertragen werden können, sind in Deutschland erhobene Daten von besonderem Wert [21].

In dieser Studie wird eine Übersicht über die Versorgungssituation der Lupuserkrankten in Rheinland-Pfalz und dem Saarland geschaffen, insbesondere in Bezug auf erkrankungsrelevante Items wie die Anzahl der behandelten Patienten, Hauptsymptomatik, Medikation und Remission.

## Studiendesign und Untersuchungsmethoden

Die Datenerhebung erfolgte von August 2020 bis April 2021. Es wurden Fragebögen an Rheumatologen, Nephrologen, Neurologen, Dermatologen und Hausärzte in Rheinland-Pfalz und dem Saarland versendet.

Die Fragebögen wurden zunächst per Fax und durch den Newsletter der kassenärztlichen Vereinigung Rheinland-Pfalz (KV-RLP) an 1546 Empfänger geschickt, wobei entweder per Mail oder per Fax geantwortet werden konnte. Die Empfänger wurden von April bis Oktober 2020 über eine Internetrecherche getrennt nach Facharztrichtungen und Praxen beziehungsweise Kliniken ermittelt. Dazu wurde der Arztfinder der jeweiligen kassenärztlichen Vereinigungen genutzt sowie verschiedene Arztsuche-Webseiten wie Jameda oder Medfuehrer.

Aufgrund geringer Rückläufe von nur 28 Antworten (1,8 %) in dieser ersten Runde wurde der Fragebogen auf eine Seite mit den zehn zu priorisierenden Fragen gekürzt sowie im Verlauf digitalisiert und vergütet. Abgefragt wurden unter anderem die Anzahl der behandelten SLE-Patienten, Hauptsymptome, Therapieregime, Remission, Komorbiditäten und GC-Dosen. In einem Freifeld konnten Verbesserungsvorschläge genannt werden.

Dieser digitale Fragebogen wurde im Quartal 1/2021 in drei Runden per E‑Mail an 1219 Praxen (niedergelassene Fachärzte) und Zentren (Kliniken mit Ambulanzanbindung) versendet. Der Rücklauf betrug 118 Antworten (9,7 %), davon 57 mit SLE-Patienten (48,3 %). Insgesamt wurden Rückmeldungen über 635 Patienten gegeben.

Die Auswertung der Daten erfolgte deskriptiv, um einen Überblick über die SLE-Versorgungssituation in den genannten Bundesländern zu erhalten.

## Ergebnisse

**Abb. 1:**
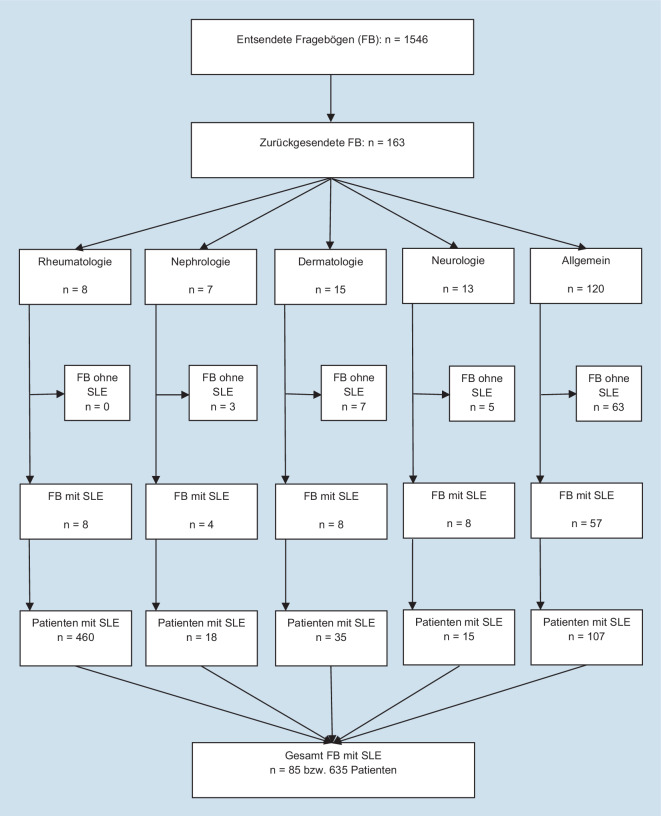
Verlaufsbaum der Versand- und Rücklaufquote der teilnehmenden Praxen und Zentren. *SLE* Lupus erythematodes

Es flossen insgesamt 163 ausgefüllte Fragebögen von 1546 versendeten in die Auswertung ein, mit folgender Aufteilung: 4 rheumatologische Zentren, 4 rheumatologische Praxen, 7 nephrologische Praxen, 1 dermatologisches Zentrum, 14 dermatologische Praxen, 5 neurologische Zentren, 8 neurologische Praxen sowie 120 hausärztliche Praxen. Die Rücklaufquoten der finalen Versandrunde variieren dabei von ca. 8 % (Nephrologie) bis 12 % (Dermatologie). Von den insgesamt 163 Antwortenden behandelten 85 in ihren Einrichtungen insgesamt 635 an SLE erkrankte Patienten (s. Abb. 1), davon 457 Patienten aus Rheinland-Pfalz und 178 aus dem Saarland.

Die weiblichen Patientinnen (84 %) waren im Durchschnitt 50,8 Jahre alt, die männlichen Patienten (16 %) waren im Durchschnitt 53,2 Jahre alt. Es stellte sich ein Verhältnis von 5:1 weibliche zu männliche Patienten heraus.

Aufgrund der Anonymität der Fragebögen ist nicht ausgeschlossen, dass einige Patienten von mehreren antwortenden Fachärzten behandelt wurden und damit als Teil mehrerer Patientenkollektive dokumentiert wurden.

### Hauptsymptome

Bei den angegebenen Hauptsymptomen dominierten im Mittel der Fachärzte Arthralgien (64 %) und Fatigue (61 %), gefolgt von Myalgien (42 %), Hautveränderungen (38 %) und dem Raynaud-Phänomen (35 %). Es fiel auf, dass einige Fachärzte vor allem die Symptome ihrer jeweiligen Fachrichtung beschrieben. Durch die neurologischen Fachärzte wurden neben neurologischen und neuropsychiatrischen Symptomen auch Myalgien und Arthralgien besonders häufig beschrieben. Die antwortenden Hausärzte lagen mit der beschriebenen Häufigkeit der Hauptsymptome etwa im Durchschnitt der anderen Fachärzte.

### Medikamentöse Behandlung und Therapieerfolg

Bei der Analyse der Antworten aller Ärzte fällt auf, dass der medikamentöse Fokus vor allem auf AMM und GC lag. Dies entspricht in Grundzügen den Empfehlungen der Literatur bezüglich Schub- und Dauertherapie. In der Nutzung der AMM unterschieden sich die Rheumatologen stark von den anderen Fachärzten. Während die Rheumatologen im Mittel bei 81 % ihrer Patienten Antimalariamittel einsetzten, erhielten dies im Durchschnitt nur 35 % der SLE-Patienten der anderen fachärztlichen Gruppen. Besonders in der Dermatologie wurde mit 27 % einem sehr geringen Anteil an Patienten AMM verschrieben. Diese Zahlen liegen weit hinter den angestrebten bis zu 100 % AMM-Einsatz zurück.

Auf der anderen Seite erhielten 4 % der Patienten dauerhaft mehr als 10 mg/d Prednisolon-Äquivalent (PÄ) sowie 22 % zwischen 5 und 10 mg/d, womit 26 % der Patienten dauerhaft mehr als die empfohlenen 5 mg oder weniger PÄ pro Tag einnehmen.

Immunsuppressiva zum Einsparen von GC wurden hingegen insgesamt nur wenig verschrieben (im Mittel erhielten 19 % MTX, 14 % AZA, 11 % MMF und 18 % Belimumab). Auffallend war hierbei, dass vor allem MTX und Belimumab hauptsächlich durch Rheumatologen verschrieben wurden (22 % MTX, 24 % Belimumab).

Unter der angegebenen Therapie befanden sich 76 % in Remission (Definition hier: „keine Symptomatik“), darunter 5 % therapiefrei, 64 % unter immunsuppressiver Therapie und 8 % unter Kortisonmonotherapie.

Eine Übersicht zu Hauptsymptomatik, medikamentöser Therapie und Remission findet sich in Tab. 1.

**Tab. 1:** Gegenüberstellung der Angaben der Ärzte

	Rheumatologen (*N* = 460) (in %)	Nephrologen (*N* = 18) (in %)	Neurologen (*N* = 35) (in %)	Dermatologen (*N* = 15) (in %)	Hausärzte (*N* = 107) (in %)
Hauptsymptomatik	69 Arthralgien 66 Fatigue 42 Raynaud-Syndrom 38 Myalgien	73 Nierenbeteiligung 62 Fatigue 43 Vaskulitis	87 Fatigue 77 Arthralgien 75 Myalgien 67 neurologische Symptomatik	77 Hautveränderungen 27 Fatigue 26 Sicca-Symptomatik	54 Hautveränderungen 52 Arthralgien 47 Myalgien 44 Fatigue
Medikamentöse Therapie	81 AMM 30 < 20 mg PÄ 24 Belimumab 20 MTX	53 < 20 mg PÄ 36 AMM 36 ≥ 20 mg PÄ	40 < 20 mg PÄ 27 AMM 13 AZA*	78 GC 53 < 20 mg PÄ 35 AMM*	37 AMM 34 < 20 mg PÄ 28 NSAR
Remission („symptomfrei“)	75, darunter 73 unter IS-Therapie, 1 therapiefrei und 1 unter GC-Monotherapie	96, darunter 39 unter IS-Therapie, 39 unter GC-Monotherapie und 18 therapiefrei	27, alle davon unter IS-Therapie	66 , darunter 34 unter IS-Therapie, 24 unter GC-Monotherapie und 8 therapiefrei	77, darunter 47 unter IS-Therapie, 17 unter GC-Monotherapie und 13 therapiefrei

*AMM* Antimalariamittel; *MTX* Methotrexat; *PÄ* Prednisolon-Äquivalent; *NSAR* Nichtsteroidale Antirheumatika; *IS* Immunsuppressiva; *GC* Glukokortikoide; *AZA* Azathioprin

Die Differenzierung zwischen Zentren und Praxen wurde begrenzt durch die Tatsache, dass lediglich drei Patienten aus Zentren nicht-rheumatologisch betreut wurden. Daher erfolgte die Gegenüberstellung der Hauptsymptomatik und der medikamentösen Therapie lediglich zwischen rheumatologisch betreuten Patienten aus Praxen gegenüber rheumatologischen Zentren. Es zeigt sich, dass insbesondere die Fatigue deutlich häufiger in Zentren gesehen wird (siehe Tab. 2). Bezüglich der Therapie lässt sich sagen, dass alle rheumatologisch betreuten Patienten vergleichsweise häufig mit AMM behandelt werden, in Praxen sogar noch häufiger als in Zentren. Nach den AMM wird in den Zentren interessanterweise Belimumab am häufigsten eingesetzt vor Mtx. Der GC-Einsatz ist hingegen in Zentren deutlich geringer.

**Tab. 2:** Gegenüberstellung der Angaben aus rheumatologischen Praxen und rheumatologischen Zentren

	Praxen (*N* = 68) (in %)	Zentren (*N* = 392) (in %)
Hauptsymptomatik	65 Arthralgien 54 Hautveränderungen 50 Myalgien 47 Fatigue	73 Arthralgien 68 Fatigue 44 Raynaud-Phänomen 40 Myalgien
Medikamentöse Therapie	90 AMM 61 < 20 mg PÄ 26 MTX	79,5 AMM 28,6 < 20 mg PÄ 18,7 MTX

*AMM* Antimalariamittel; *MTX* Methotrexat; *PÄ* Prednisolon-Äquivalent

### Komorbiditäten

Die am häufigsten angegebenen Komorbiditäten waren das Fibromyalgiesyndrom (FMS, 26 %), Depressionen (24 %) und kardiovaskuläre Schädigungen (21 %). Die Prävalenz der Osteoporose lag mit 10 % etwa im Durchschnitt der deutschen Bevölkerung [17]. Am häufigsten unterschieden sich die fachärztlichen Gruppen im Hinblick auf das FMS, welches 39 % der Neurologen, 57 % der Nephrologen und 63 % der Rheumatologen als häufige Komorbidität ankreuzten. Stark korreliert und nur unter den Neurologen mit 8 % vom FMS abweichend berichteten diese Fachärzte auch von vermehrten Depressionen unter ihren SLE-Patienten. Unter den Dermatologen berichteten nur 13 % von FMS und Depressionen als Komorbiditäten ihrer SLE-Patienten. Die Rheumatologen hingegen unterschieden sich in der Angabe der Komorbiditäten teilweise deutlich von ihren Kollegen. Kardiovaskuläre Komorbiditäten stachen mit 75 % deutlich hervor, ebenso Anämien, Osteoporose und Adipositas mit je 38 %.

### Bewertung der Versorgungssituation

Bei einer Bewertung der Versorgungssituation von Patienten mit SLE mit einer Schulnote von eins bis sechs wurde in Rheinland-Pfalz eine Durchschnittsnote von 3,2 vergeben, während die saarländischen Ärzte im Durchschnitt eine Note 2,8 erteilten. Ärzte aus Praxen vergaben die Durchschnittsnote 3,1, solche aus Zentren die Durchschnittsnote 3,0.

### Verbesserungsvorschläge zur Versorgungssituation im Freitext

Ein Freitextfeld für Verbesserungsvorschläge ließen 43 % der Antwortenden leer. Von den erbrachten Kommentaren waren 3 % als positiv zu werten. 50 % beschäftigten sich mit dem Mangel an Rheumatologen bzw. der Schwierigkeit, Termine bei einem Rheumatologen zu bekommen. Insgesamt 17 % wünschten sich mehr Fortbildung oder Aufklärung, 12 % mehr Vernetzung und Kommunikation und 18 % der Kommentare schlugen sonstige Verbesserungsmaßnahmen vor.

## Diskussion

In der vorliegenden Arbeit wurden erstmals gezielt Informationen über die Versorgungssituation von Patienten mit SLE in Rheinland-Pfalz und dem Saarland erhoben. Die in dieser Studie erhobenen Daten geben wertvolle Einblicke in Epidemiologie, Symptomatik, Therapie und Behandlungserfolge aus Sicht der niedergelassenen Kollegen.

Das Verhältnis von weiblichen zu männlichen SLE-Patienten unter den rückläufigen Fragebögen betrug 5:1. Dies entspricht in etwa dem von Brinks et al. (2014) ermittelten Verhältnis aus ihrer Untersuchung deutscher SLE-Patienten von 4:1, unterschreitet jedoch erheblich das häufig in der Literatur angegebene Verhältnis von 9:1 [4].

Unter den Hauptsymptomen dominierten im Mittel der Fachärzte Arthralgien und Fatigue mit je etwa 60 %, Hautveränderungen zeigten etwa 38 % der Patienten. In der Literatur sind besonders muskuloskelettale Symptome sowie Fatigue mit je nach Studie bis zu etwa 90 % deutlich häufiger beschrieben [17, 19]. An diese Zahlen näherten sich die Antworten der Rheumatologen mit 69 % Arthralgien und 66 % Fatigue an. Insgesamt kann jedoch keine Tendenz hin zu deutlich höheren Symptomraten unter rheumatologisch behandelten Patienten festgestellt werden.

Eine Nierenbeteiligung beklagten 14 % der Patienten des befragten Kollektivs. Diese Zahl liegt deutlich unter den ermittelten 22 % eines anderen befragten deutschen Kollektivs von Fischer-Betz et al. [15]. Fieber/Schwäche gaben im befragten Kollektiv 24 % der Patienten an, was deutlich weniger ist als in einem 200 Patienten umfassenden Kollektiv von Sloan et al. (2020), in dem etwa 78 % von längerfristigem Fieber berichteten [27]. Das Raynaud-Phänomen wurde mit 35 % vergleichsweise häufig genannt, reichte damit aber nicht an die Häufigkeit in einer Kohorte von Nyman et al. (2020) heran (ca. 52 %) [23].

Insgesamt fällt eine Fokussierung auf den jeweiligen Fachbereich auf. Womöglich werden hierdurch einige fachfremde Symptome, wie beispielsweise Hautveränderungen nicht dermatologisch betreuter Patienten, übersehen oder übergangen.

Zur medikamentösen Behandlung ist zunächst zu sagen, dass die Empfehlungen der Literatur in Bezug auf die Basistherapie eine große Übereinstimmung aufweisen: Prinzipiell sollte jeder SLE-Patient Antimalariamittel erhalten [11]. Glukokortikoide haben eine große Bedeutung im Schub, langfristig sind sie aber vor allem schädlich und sollten wo immer möglich durch den Einsatz von Immunsuppressiva oder Auslassversuche eingespart werden. Im befragten Kollektiv dieser Arbeit war der Einsatz von AMM im Vergleich zur Literatur unterdurchschnittlich. In einer Analyse von Daten der Kerndokumentation 2018 nahmen 67 % aller SLE-Patienten AMM ein, ein Wert, den unter den Befragten nur die Fachärzte der Rheumatologie mit 81 % übertrafen [8]. Die anderen Fachärzte verschrieben im Mittel nur 35 % ihrer Patienten AMM. Auch der Einsatz von Glukokortikoiden als zweiter großer Säule bei im Mittel 46 % der Patienten entspricht den Erwartungen. Dieser unterschritt jedoch den in der Kerndokumentation beschriebenen Wert von 62 % und auch den anderer Kohorten von bis zu 88 % [2].

Es könnte der vermehrte Einsatz von Immunsuppressiva sein, der den Rheumatologen den unterdurchschnittlichen Einsatz sehr hoch dosierter GC (≥ 20 mg/d) von 1 % (verglichen mit 4 % über alle Fachärzte hinweg) ermöglichte. Dieser wiederum war unter den Nephrologen mit 36 % um ein Vielfaches erhöht. Eine mögliche Erklärung hierfür könnte im hohen Anteil renaler Schübe liegen, wobei die in der Literatur empfohlene Therapie aus MMF und/oder niedrig dosiertem Cyclophosphamid besteht und GC möglicherweise nicht erforderlich sind [10, 12]. Ein insgesamt vergleichsweise hoher Einsatz von Immunsuppressiva durch die Nephrologen könnte die Aktivität der SLE-Manifestation unterstreichen.

Dieser war in der Dermatologie nicht zu sehen. Der Einsatz von Immunsuppressiva lag in dieser fachärztlichen Gruppe weit unter dem Durchschnitt, während 78 % der Patienten Glukokortikoide verschrieben wurden. Ein möglicher Grund könnte der routinierte Umgang mit GC-haltigen Externa in der Dermatologie sein.

Der dauerhafte (länger als sechs Monate erfolgte) Einsatz höher dosierter Glukokortikoide (> 5 mg/d) lag im Durchschnitt aller Fachärzte bei 26 %. Auffällig ist, dass die Rheumatologen als einzige fachärztliche Gruppe bei vielen Patienten dauerhaft Glukokortikoide in Dosierungen unter 5 mg/d einsetzten, wobei die höhere Quote mit Beobachtungen aus der Literatur übereinstimmt [18]. Sie erreichten in dieser Kategorie eine Quote von 33 %, während sonst nur die Allgemeinmediziner (11 %) und die Dermatologen (8 %) überhaupt dauerhaft niedrig dosierte Glukokortikoide verschrieben. Diese nebenwirkungsärmere Langzeittherapie wäre gegenüber höher dosierten GC in deutlich mehr Fällen wünschenswert.

Beim Blick auf die Remissionsraten der behandelten Patienten – wobei remittiert im Fragebogen als symptomfrei definiert wurde – fällt auf, dass sich 75 % der rheumatologisch behandelten Patienten in einer Form der Remission befanden. Die Unterscheidung nach Therapieform zeigte auf, dass 73 % unter Immunsuppression symptomfrei waren, was auf schwierige Fälle hinweisen könnte, die leitliniengerecht therapiert werden. Die Nephrologen wiesen mit 96 % eine noch höhere Remissionsrate auf, wobei in diesem Fall auffällt, dass 39 % der Patienten symptomfrei unter Kortisonmonotherapie waren, eine Behandlung entgegen den Empfehlungen der Literatur. Auch unter den dermatologisch und allgemeinmedizinisch betreuten Patienten waren einige unter Kortisonmonotherapie symptomfrei (24 % bzw. 17 %). Unter den rheumatologisch behandelten Patienten traf dies nur für 1,1 % zu. Auffällig ist zudem, dass sich unter den neurologisch behandelten Patienten mit 27 % weit unterdurchschnittlich viele in Remission befanden. Ob dies mit dem geringen Einsatz an AMM (27 %) und Immunsuppressiva (13 % AZA, 0 % MTX, 0 % MMF) oder mit der speziellen neurologisch, neuropsychiatrischen Manifestation zusammenhängt, lässt sich bei der geringen Anzahl an neurologischen Rückläufen nur mutmaßen. Weitere Forschungsarbeiten wären hier interessant. Symptomfrei ohne Therapie waren mit insgesamt 5 % nur wenige Patienten.

Die Begrifflichkeit der Remission kann durchaus kritisch gesehen werden. Obwohl mit den „DORIS-Kriterien“ (definition of remission in SLE) eine objektive Bewertung möglich ist, können die Interpretation und das Empfinden durchaus divergieren. Im Abgleich mit der Literatur zeigt sich, dass sich aus ärztlicher Sicht der Großteil ihrer SLE-Patienten in Remission befindet. Betrachtet man jedoch die Therapie bei remittierten und nicht remittierten Patienten, zeigt sich eine vergleichbare Therapie, da nach ärztlicher Einschätzung die Remission häufig einen Zustand beschreibt, in dem man nichts Weiteres mehr tun muss oder kann [22]. Zusammenfassend ist nicht auszuschließen, dass es Unterschiede zwischen den Facharztgruppen gibt, jedoch ist dies aufgrund des Studiendesigns nicht prüfbar.

Der Vergleich zwischen rheumatologischen Praxen und rheumatologischen Zentren zeigt insgesamt vergleichbare Quoten an Symptomen und Medikamenteneinsatz. An Zentren wird Belimumab deutlich häufiger eingesetzt (27 % vs. 4 % in Praxen), was womöglich den deutlich geringeren GC-Einsatz begünstigt.

Bezüglich der Komorbiditäten fällt auf, dass die rheumatologischen Fachärzte einige deutlich häufiger nannten als ihre Kollegen. Dies könnte auf schwerer erkrankte Patienten oder aber höhere Aufmerksamkeit gegenüber diesen Komorbiditäten zurückzuführen sein, von denen einige direkt mit dem SLE zusammenhängen.

Die Auswertung der Kommentare zeigte, dass sich der weitaus größte Teil mit der Forderung nach mehr niedergelassenen Rheumatologen, nach mehr rheumatologischen Zentren, leichterem Zugang und schnellerer Terminvergabe befasste. Auch die Kommunikation sollte verbessert werden, möglicherweise durch Netzwerke, wofür ebenfalls die Zeit der rheumatologischen Fachärzte und damit die Anzahl derselben erhöht werden müsste. All dies deckt sich mit Beobachtungen in der Literatur, dass in Deutschland eine massive Unterversorgung mit Rheumatologen herrscht. Daten von Zink et al. stellten heraus, dass 2016 in Rheinland-Pfalz und dem Saarland nur 0,8 Rheumatologen anstelle eines errechneten Bedarfs von 2 pro 100.000 Erwachsenen vorhanden waren und deutschlandweit ca. 18 % zu wenig stationäre rheumatologische Betten zur Verfügung standen [29]. Nicht überraschend war in dem Sinne die Erkenntnis, dass 45 % der befragten Patienten einer Kohorte von Danoff-Burg und Friedberg 2009 unzufrieden waren mit der Kontinuität der Betreuung und der Menge an Zeit, die sie mit Ärzten verbrachten, während gleichzeitig gezeigt werden konnte, dass mehr klinische Versorgung die Krankheitsaktivität, den Schaden und die Health-Related Quality of Life (HRQoL) positiv beeinflusst [7, 18].

Auch die in den Kommentarfeldern häufig gestellte Forderung nach Fortbildung und Aufklärung deckt sich mit den bekannten Herausforderungen. Eine bereits in der Literatur beschriebene unzureichende Umsetzung der Leitlinien, die sich beispielsweise in hoch dosierten GC-Dauertherapien widerspiegelt, geringe Verschreibung von AMM oder zu geringe Quoten einiger zum Teil unspezifischer oder fachfremder Hauptsymptome lassen auf einen Fortbildungsbedarf zum Thema SLE schließen [30]. Insbesondere hinsichtlich der Therapie zeigte sich eine deutlich leitliniengerechtere Behandlung seitens der rheumatologischen Fachkollegen.

In diesem Zusammenhang ist die aktuelle Studie von Aringer et al. (2021) zu erwähnen, die die schwache Repräsentanz der Rheumatologie in der medizinischen Lehre an deutschen Universitäten bemängelt [3]. Die Autoren empfehlen verbindliche Lernziele, die in mindestens 6 Doppelstunden Pflichtvorlesung in internistischer Rheumatologie vermittelt werden sollen. Diese können, verknüpft mit einer Integration der Lerninhalte in relevante universitäre Prüfungen, „das Wissen um rheumatische Erkrankungen und damit die Versorgung der Menschen verbessern, die unter ihnen leiden“ [3].

Auch eine, ebenfalls in vielen Kommentaren geforderte, weitere Vernetzung und intensivierte interdisziplinäre Kommunikation wird in der Literatur angestrebt, unter anderem um schwerwiegende Komorbiditäten zu erkennen oder auszuschließen [10, 19]. Die Ergebnisse der Symptomabfrage zeigten beispielsweise einen sehr engen Blick auf das jeweilige Fachgebiet mit zum Teil deutlich niedrigeren Quoten bei fachfremden Hauptsymptome. Eine interdisziplinäre Vernetzung und Behandlung könnte solche Unzulänglichkeiten ausgleichen.

## Schlussfolgerung

Die landesweite Datenerhebung in Rheinland-Pfalz und dem Saarland ermöglicht interessante Einblicke in die Versorgungsrealität von SLE-Patienten jenseits von Theorie und Leitlinien. Erstmals konnten spezifische Informationen zu Epidemiologie, Hauptsymptomen, medikamentösen Therapien und Komorbiditäten in den beiden Flächenländern gewonnen werden. Trotz aller Einschränkungen einer fragebogenbasierten Erhebung liefert die Studie wichtige Ansätze für dringende Optimierungen.

## Fazit für die Praxis

Für eine Verbesserung der Versorgungssituation von SLE-Patienten werden basierend auf den Ergebnissen der besprochenen Studie folgende Vorschläge gemacht: Es wird eine Änderung der Bedarfsplanung für den Ausbau der rheumatologischen Sitze und Anreize zur rheumatologischen Niederlassung empfohlen. Zudem wäre ein Ausbau des rheumatologischen Lehrangebotes an medizinischen Fakultäten sinnvoll. Mehr Aufklärung und Fortbildung aller Fachärzte zu rheumatologischen Krankheitsbildern sowie die Förderung der interdisziplinären Kommunikation und Vernetzung werden als zielführend erachtet. Abschließend empfehlen wir die Erstellung vereinfachter Leitfäden zur Diagnostik und Therapie des systemischen Lupus Eerythematodes (SLE).

## Footnotes

1 Der besseren Lesbarkeit halber wird in dieser Arbeit das generische Maskulinum verwendet, wobei stets alle Geschlechter gemeint sind.
